# Does equity in healthcare spending exist among Indian states? Explaining regional variations from national sample survey data

**DOI:** 10.1186/s12939-017-0517-y

**Published:** 2017-01-14

**Authors:** Rinshu Dwivedi, Jalandhar Pradhan

**Affiliations:** 1 Research Scholar Department of Humanities and Social Sciences, National Institute of Technology, Rourkela, Odisha 769 008 India; 2 Department of Humanities and Social Sciences, National Institute of Technology, Rourkela, Odisha 769 008 India

**Keywords:** Equity, Inequalities, Healthcare utilisation, GLRM, OOPE

## Abstract

**Background:**

Equity and justice in healthcare payment form an integral part of health policy and planning. In the majority of low and middle-income countries (LMICs), healthcare inequalities are further aggravated by Out of Pocket Expenditure (OOPE). This paper examines the pattern of health equity and regional disparities in healthcare spending among Indian states by applying Andersen’s behavioural model of healthcare utilization.

**Methods:**

The present study uses data from the 66^th^ quinquennial round of Consumer Expenditure Survey, of the National Sample Survey Organization (NSSO), conducted in 2009–10 by Ministry of Statistics and Programme Implementation (MoSPI), Government of India (GoI). To measure equity and regional disparities in healthcare expenditure, states have been categorized under three heads on the basis of monthly OOPE i.e., Category A (OOPE > =INR 100); Category B (OOPE between INR 50 to 99) and Category C (OOPE < INR 50). Multiple Generalised Linear Regression Model (GLRM) has been employed to explore the effect of various socio-economic covariates on the level of OOPE.

**Results:**

The gap in the ratio of average healthcare spending between the poorest and richest households was maximum in *Category A* states (richest/poorest = 14.60), followed by *Category B* (richest/poorest 11.70) and *Category C* (richest/poorest 11.40). Results also indicate geographical concentration of lower level healthcare spending among Indian states (e.g., Odisha, Chhattisgarh and all the north-eastern states). Results from the multivariate analysis suggest that people residing in urban areas, having higher economic status, belonging to non-Muslim communities, non-Scheduled Tribes (STs), and non-poor households spend more on healthcare than their counterparts.

**Conclusions:**

In spite of various efforts by the government to reduce the burden of healthcare spending, widespread inequalities in healthcare expenditure are prevalent. Households with high healthcare needs (SCs/STs, and the poor) are in a more disadvantaged position in terms of spending on health care. It has also been observed that spending on healthcare was comparatively lower among backward or isolated states. No doubt, the overall social security measures should be enhanced, but at the same time, looking at the regional differences, more priority should be assigned to the disadvantaged states to reduce the burden of OOPE. It is proposed that there is need to increase government spending, especially for the disadvantaged states and population, to minimise the burden of OOPE.

## Background

Health equity has been an important policy issue since the Alma-Ata Declaration of the World Health Organization (WHO). Since then all countries have been making efforts to reduce health inequities. Concerns have been expressed regarding the quality and availability of healthcare services in low and middle-income countries (LMICs) [1, 2]. Accessibility and affordability of healthcare services are among the major healthcare challenges faced by developing countries [3–5]. Financial barriers are key limitations to access healthcare services in LMICs since Out-of-Pocket Expenditure (OOPE)^1^ constitutes a significant proportion of healthcare expenditure [6–8]. Globally, about 1.3 billion people are deprived of access to effective and affordable health care. Majority of households spend more than 40% of their household income on medical treatment [9, 10].

Possibilities are high of many households residing in LMICs being pushed into poverty when faced with high medical expenses, especially when it is combined with loss of income due to adverse health outcomes [11, 12]. Measures to promote financial protection through universal health coverage (UHC) strategize to ensure that people would have access to health services without risking financial impoverishment [13–15]. Health systems in many LMICs are financed through key sources such as, taxation, social security schemes, private health insurance and OOPE [15].

More than half of the world’s population does not have access to formal social protection schemes [16, 17]. Majority of the households who are unable to pay for using healthcare services either do not seek care or resort to short-term coping strategies such as minimizing food expenses, using past savings, and removing children from school to manage the financial shortfall [18–20]. Short-term coping strategies may result in higher cost in the long run, leading to impoverishment and poverty. These households are not captured in poverty estimates, as high healthcare cost raises their expense above the threshold level and they are considered non-poor [14].

Literature shows that the level of health payments also differs significantly with variations in certain specific characteristics of the households. Poor and disadvantaged sections of the population face more financial risks and need better financial mechanisms to avail healthcare services [21, 22]. Literature also indicates differentials in healthcare spending among various regions as well as segments of population [23, 24].

### Indian scenario

Equity and justice in healthcare payment are integral parts of health policy draft in India [25, 26]. Healthcare system in India is highly privatized and the main source of financing is OOPE [27, 28]. OOPE does not provide any financial protection; as a result, such households incur heavy expenses in availing healthcare services [29]. Expenditure on healthcare pushes a large number of families into poverty in India as they do not have sufficient spending power due to low level of income or sometimes, no fixed source of income [30–32].

In India, more than 90% of the workforce, especially people who belong to socially and economically underprivileged sections of society, is engaged in informal economic activities.^2^ As insurance facilities are available only to the workforce in the formal sector, majority of such households are not covered under any social protection scheme [33, 34]. In the case of ill health, these households have to spend from their own pockets. Inadequate provisioning of health care facilities, coupled with a highly privatized health sector, further worsens the financial status of the poor and marginalized groups of the population [35, 36]. It leads them into financial catastrophe and further deepens existing poverty [36–39].

Level and pattern of OOPE are determined on the basis of socio-economic affiliations in India. Differences also prevail in the health spending and level of socio-economic inequalities among different Indian states. Different variables such as, socio-economic status, class, religious affiliation, place of residence, gender and age are used to classify the population [35, 40, 41]. Among all these factors, economic status and caste affiliation are considered most important classificatory variables for analyzing socio-economic inequalities in health and health care expenditure in India [42–45]. Socio-economic inequalities are highly unfavourable for healthcare, especially when society is more diversified, multi-ethnic, overpopulated and undergoing significant but unequal economic growth [46].

There is evidence of wider inter-state differentials in public spending and health infrastructure across Indian states. The level of public spending on health in few of the backward states such as, Bihar, Jharkhand, and Odisha, is very low in comparison to Kerala, Punjab and other developed states [47]. Studies also indicate increasing interstate inequalities in health spending in recent years. The difference between per capita OOPE among developed states such as, Kerala and Punjab, and backward states such as, Jharkhand, Chhattisgarh and Orissa, has increased, leading to greater divergence between these states [48, 49].

Only a limited number of studies are available in the Indian contexts which have tried to examine the level of equity and regional variations in healthcare spending, by categorizing Indian states. We have classified the states on the basis of OOPE into three categories i.e., high, medium and low level of OOPE, and examined the pattern of inequalities in health spending among these states. This paper makes an effort to examine the pattern of health equity and regional disparities in healthcare spending among the Indian states by applying Andersen’s behavioural model of healthcare utilization.

## Data and methods

### Data

Data is used from the 66^th^ quinquennial round of the Consumer Expenditure Survey (CES), National Sample Survey Organisation (NSSO), conducted by the Government of India (GoI) in 2009–10. The present study uses schedule type-1 questionnaire, which covers 100,855 households, of which nearly 59% are located in rural areas. We have taken into consideration consumption expenditure as a measure of healthcare spending. As there are noticeable differences between developed and developing countries regarding the nature and extent of formal employment, level of poverty and health indicators, consumption is used as the standard measure of overall material well-being among developing countries. In developing countries, only a limited segment of the population is employed in the formal sector while a majority of the population is engaged in the informal sector without any fixed source of income. Under such circumstances, consumption is better measured than income for poor families, and is less vulnerable to under-reporting bias. There are also conceptual and economic reasons to prefer consumption measure rather than income because consumption is a more direct measure of material well-being. Spending on healthcare not only depends on income but also on sources such as, savings, borrowings and other sources. Available literature tends to support the consumption method, signifying that consumption should be used to assess spending on healthcare and to set other benefit criteria [6, 8, 40, 50].

The CES rounds contain information on household consumption expenditure for both food and non-food items. Healthcare expenses are covered under non-food section and can be broadly classified into institutional and non-institutional health expenditure. The recall period is 365 days for institutional expenses and 30 days for non-institutional expenses. This study uses data on OOPE for both institutional (inpatient)^3^ and non-institutional (outpatient)^4^ expenditure by using a 30-day recall period. Institutional expenditure was available for a 365-day recall period; so, for the purpose of the study, it has been converted into 30-day recall period (see Appendix). The approach to measure OOPE for healthcare payments has been adopted from Wagstaff and Doorslaer [40]. In addition to medical expenditure, information is also available on socio-economic and demographic characteristics of the households. The questionnaire contains information about individuals and households such as, place of residence (urban/rural), religious affiliation (Hinduism, Islam, Christianity, Sikhism, Jainism, Buddhism, Zoroastrianism and others), social group^5^ (Scheduled Caste (SC)^6^, Scheduled Tribe (ST),^7^ Other Backward Caste (OBC)^8^and Others).

### Methods

States has been classified into three categories i.e., Category A (Higher OOPE), Category B (Middle-level OOPE) and Category C (lower OOPE) states. Though it is ideal to categorize the states on the basis of mean/median values of OOPE, our objective was to look into the equity and efficiency aspects among the states with higher, lower and middle levels of OOPE. First, we categorized the states on the basis of the level of monthly OOPE in three categories i.e., Category A (OOPE > =INR 100); Category B (OOPE between INR 50 to 99) and Category C (OOPE < INR 50). Next, mean OOPE was calculated for the three categories of states (A, B, and C), by selected socio-economic covariates. Lastly, we employed multiple Generalized Linear Regression Model (GLRM) to explore the effect of various socio-economic covariates on the level of OOPE. Our outcome variable OOPE was usually non-parametric and positively skewed with influential outliers. Traditional Ordinary Least Square (OLS) regressions with log-transformation cannot accurately capture the skewness in the data. It can provide inferences in the natural units of average healthcare expenditure. [51]. GLRM can flexibly handle the skewed datasets and reduce the problem of outcome transformation [52, 53]. We have employed GLRM with gamma distribution and log link function to examine the various determinants of OOPE [54].

### Dependent variable

The dependent variable for the study is OOPE among all Indian states and UTs (both rural and urban).

### Independent variable

The independent variables are: residence (Rural /Urban), economic status (wealth quintile), religious group (Muslims and others), and social group (Scheduled Tribe and others). For measurement of the economic status, we have used the households quintile based ranking on MPCE, separately for both urban and rural areas. In the Indian social setup, caste is broadly divided into different groups such as, SCs, STs, OBCs and others category. We have re-classified the caste groups into two i.e., STs and Others (Including SCs, OBCs and others), and religious categories, into Muslims and non-Muslims, (Non-Muslims include Hindus, Christians, Sikhs, Buddhism, Jainism, Zoroastrianism and other smaller religious communities).

### Conceptual framework

This paper also tries to apply Andersen’s behavioural model of healthcare utilization which was initially developed in the late1960s [55, 56]. As per this model, use of healthcare services by the households is a function of their predisposition to use services, factors which allow them to use these services and their need to use these services. This model categorizes the use of health care services as a function of three elements - *predisposing, enabling* and *need* factors. *Predisposing* factors include demographic variables such as, residence and socioeconomic status (social and religious group). Enabling factors include the wealth or economic status of the population; level and pattern of OOPE, which is an indicator of health status, are considered *need* factors.

**Fig. 1:**
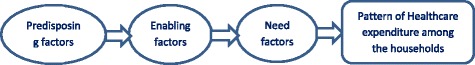
Study framework adapted from Anderson’s Model. Source: Partially adapted from Andersen’s behavioural model [55]

Based on Andersen’s model, we have also classified the factors determining the use of healthcare services in three categories: *predisposing, enabling* and *need* factors (Fig. 1). We have included place of residence, religious and social affiliation as *predisposing* factors; economic status has been considered an *enabling* factor while the level of health spending has been taken into consideration as a *need* factor.

## Results

### Inter-state differentials in OOPE

Out of 100,855 households, 16,887 (16.74%) are in *Category A* states, 57,829 (57.34%) are in *Category B* and 26,139 (25.92%) are in *Category C.* States have been categorized into three groups on the basis of average monthly OOPE i.e., low, medium and high OOPE (Table 1).

**Table 1:** Categorization of all Indian states by mean OOPE (NSSO, CES-2009-10)

Category	Mean OOPE (INR)	States	Number of households (%)
A	100 or more	Punjab, Chandigarh,Maharashtra, Goa, Kerala, Pondicherry	16,887 (16.74)
B	50 to 99	Karnataka, Delhi, Madhya Pradesh, Rajasthan, J&K, Andaman and Nicobar, Uttar Pradesh, Tripura, West Bengal, Andhra Pradesh, Gujarat, Uttarakhand, Lakshadweep, Haryana, Tamil Nadu, Himachal Pradesh	57,829 (57.34)
C	Below 50	Dadra Nagar Haveli, Sikkim, Nagaland, Assam, Manipur, Daman &Diu, Meghalaya, Mizoram, Bihar, Jharkhand, Chhattisgarh, Orissa, Arunachal Pradesh	26,139 (25.92)

Source: Compiled from NSS CES Report (2009–10); computed by author using the unit level data records of CES 2009–10

**Fig. 2:**
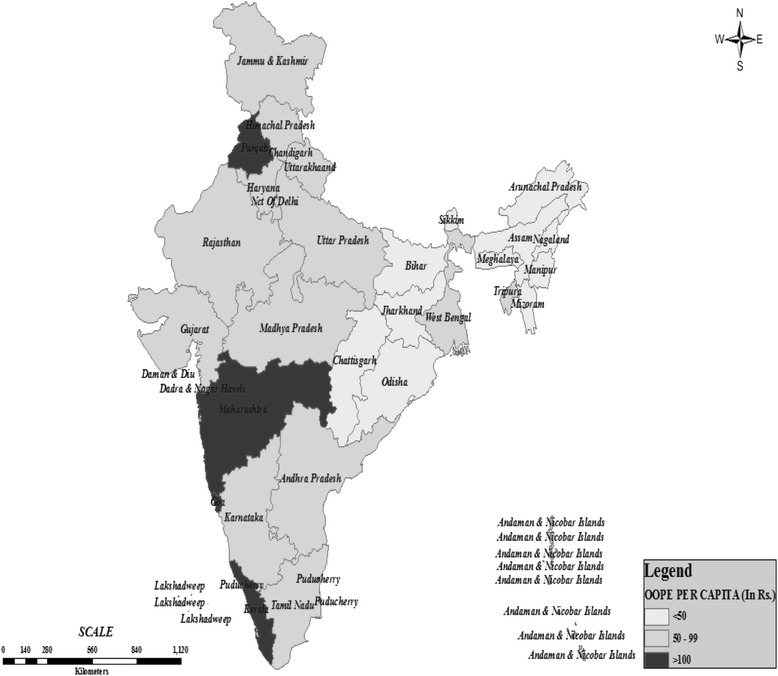
Geographical concentration of the states on the basis of average OOPE

Figure 2 shows that average monthly per capita healthcare expenditure was the highest in states such as, Punjab, Chandigarh, Maharashtra, Goa, Kerala and Pondicherry (*Category A*). *Category B* states are more concentrated towards southern India such as, Karnataka, Andhra Pradesh, Tamil Nadu, Andaman and Nicobar, Lakshadweep as well as northern states such as, Jammu and Kashmir (J&K), Delhi, Uttarakhand, Haryana, Himachal Pradesh, Rajasthan and Uttar Pradesh; states such as, Madhya Pradesh, Gujarat, Tripura and West Bengal are exceptions. *Category C* states are located in the north-eastern, central and southern eastern regions such as, Dadra Nagar Haveli, Daman & Diu, Sikkim, Nagaland, Assam, Manipur, Meghalaya, Mizoram, Arunachal Pradesh, Bihar, Jharkhand, Chhattisgarh and Orissa.

Majority of the north-eastern states fall in *Category C* and their average monthly expenditure on healthcare is less than INR 50. Results also indicate geographical concentration of lower level of health spending among these states (e.g., Odisha, Chhattisgarh and all the north-eastern states). The gap in terms of average monthly healthcare spending between the richest and the poorest households was the highest in *Category A* (Richest/Poorest = 14.60) states, followed by *Category B* (Richest/Poorest =11.70) and *Category C* (Richest/Poorest =11.40) (Table 2). Similarly, in *Category C* states, there was marginally higher gap among Muslims and Non-Muslims (Non-Muslims/Muslims =1.53) households in terms of healthcare spending followed by *Category A* (Non-Muslims/Muslims =1.13) and *Category B* (Non-Muslims/Muslims =1.13).As far as social group affiliation is concerned, the inequalities in terms of OOPE was nearly the same in both *Category A* (Others/ST = 2.58) and *B* (Others/ST = 2.34) states, and comparatively lower in *Category C* states (Others/ST = 1.17). The gap between urban and rural households in terms of healthcare spending was marginally higher in the *Category C* states (Urban/Rural =1.92), followed by *Category B* (Urban/Rural =1.62) and *Category A* states (Urban/Rural =1.26). Overall, in the Indian context, inequalities in terms of average monthly healthcare spending were observed between the richest and the poorest (Richest/Poorest =13.19), STs and Others (Others/ST = 2.50), rural and urban (Urban/Rural =1.49) households, and between Muslims and Non-Muslims (Non-Muslims/Muslims =1.11).

**Table 2:** Interstate differentials in mean OOPE by residence, economic status, religion and social group

	Categories of states			
Covariates	Category A	Category B	Category C	All India
Residence				
Rural	112.82	56.93	27.01	57.02
Urban	142.71	92.35	51.9	84.86
(Ratio Urban/Rural)	1.26	1.62	1.92	1.49
Economic status				
Poorest	19.60	19.75	16.28	18.68
Second poorest	47.92	46.25	28.51	43.22
Middle	72.15	71.92	42.20	67.96
Second Richest	123.27	94.98	62.88	97.26
Richest	286.23	230.94	185.60	246.27
(Ratio Richest/Poorest)	14.60	11.69	11.40	13.19
Religious affiliation				
Muslims	99.44	60.55	20.70	58.52
Non-Muslims (Including all others)	112.16	65.00	31.73	64.96
(Ratio Non-Muslims/Muslims)	1.13	1.07	1.53	1.11
Social group				
ST	51.20	31.40	24.86	31.00
SC	64.54	60.94	27.67	55.67
OBC	128.26	64.60	34.40	66.00
Others	132.28	73.57	29.02	77.65
(Ratio Others/ST)	2.58	2.34	1.17	2.50

Source: Compiled from NSS CES Report (2009–10); computed by author using the unit level data records of CES 2009–10

### Results from multivariate analysis

The result of the multivariate analysis is presented in Table 3. In *Category A* states, the level of OOPE is significantly lower for households that belong to the lowest wealth quintile (β = -1.501, ρ = 0.001), as compared to their counterparts in *Category* B and C states. The impact of economic status on the level of OOPE was similar in the other two categories of states i.e., *Category B* (β = -1.419, ρ = 0.000) and *C* (β = -1.139, ρ = 0.000) states. Similarly, as far as social group affiliation is concerned, the level of OOPE was the lowest among ST population as compared to their counterparts in all the categories of the states i.e., *Category A* (β = -2.97, ρ = 0.001), *B* (β = -0.386, ρ = 0.000), and *C* (β = -1.281, ρ = 0.000). *Category C* states recorded lower level of spending among Muslims (β = -0.220, ρ = 0.000), and rural communities (β = -0.158, ρ = 0.000). Place of residence and religious affiliation are important predictors of healthcare spending among the *Category C* states. However, these factors do not play any significant role in determining the level of OOPE among *Category A* and *B* states.

**Table 3:** Factors associated with OOPE among the households in Indian states (*n* = 100855)

	Categories of states			
	Category A	Category B	Category C	All states
Explanatory variables	β (95% CI)	β (95% CI)	β (95% CI)	β (95% CI)
Muslims	−0.040 (-0.169, -0.089)	−0.021 (-0.071, -0.030)	−0.220 (-0.289, -0.152)***	−0.101 (-0.155, -0.047)***
Scheduled Tribe (STs)	−0.297 (-0.467, -0.127)***	−0.386 (-0.452, -0.319)***	−0.281 (-0.341, -0.221)***	−0.634 (-0.687, -0.581)***
Rural	−0.024 (-0.064, -0.112)	−0.001 (-0.040, -0.040)	−0.158 (-0.226, -0.090)***	−0.102 (-0.139, -0.065)***
Lowest wealth Quintile	−1.501 (-1.592, -1.410)***	−1.419 (-1.455, -1.382)***	−1.139 (-1.195, -1.083)***	−1.273 (-1.321, -1.225)***

Source: compiled from NSS CES Report (2009–10); computed by author using the unit level data records of CES 2009–10

*** Significant at 1% level

As per the Andersen’s model, in the Indian context, predisposing factors such as, residence, religion and social group affiliation have a significant impact on the pattern of healthcare spending among households. Households in rural areas (β = -0.102, ρ = 0.00), belonging to the Muslims community (β = -0.101, ρ = 0.00), and ST social group (β = -0.634, ρ = 0.00) have recorded significantly lower level of OOPE than their counterparts. Enabling factors indicate that spending on healthcare among population belonging to the lowest economic status was significantly lower (β = -1.273, ρ = 0.00) as compared to other economic categories.

## Discussion

Equity in healthcare is one of the important and most desired goals to be achieved for any society. Inequalities in healthcare are measured on the basis of health outcome, utilization pattern and level of OOPE, between the Non-poor/Poor, Urban/Rural, Advantaged/Disadvantaged and other socio-economic groups of the population. Healthcare financing system should focus on achieving vertical equity (households of unequal ability should be treated unequally), horizontal equity (households of the same ability should be treated equally) and progressivity in healthcare expenditure [55–57]. This paper focuses on the equity and regional perspective of healthcare expenditure among Indian states to uncover the linkages and the burden in the form of OOPE. Results indicate that burden of OOPE is not proportional among different subgroups of the population.

People residing in the urban areas, having higher economic status, belonging to non-Muslim and non-ST groups were spending significantly higher on healthcare than their counterparts. Literature also indicates that households that belong to the lower socio-economic status (Rural, STs, Muslims and lowest wealth quintile) were constantly experiencing poor health outcomes. This may be due to the fact that these people have minimum access to healthcare facilities or they were not in a position to pay for the use of healthcare services [30, 58, 59].

There is also evidence of regional disparity in terms of healthcare spending among Indian states. It is evident from the results that in *Category A* states (Kerala, Punjab, Maharashtra, Goa and Chandigarh), the average monthly expenditure on healthcare was highest followed by *Category B* and *C*. Findings from available literature also supports the fact that *Category A* states are more affluent with higher per capita income as compared to the other categories of states (*B* and *C*). *Category C* states constitute a higher share of tribal population as compared to the other two categories of states. Due to geographical isolation and inaccessibility, these states are more deprived in terms of availability of healthcare facilities [25, 48, 49].

As in previous studies in the Indian context, our study also indicates progressivity in healthcare financing among Indian states. The incidence of OOPE was higher among *Category A* states such as, Kerala, Punjab, Maharashtra, Goa, and Chandigarh. However, *Category A* states face higher burdens of Non-communicable diseases (NCDs), causing them to spend more on healthcare, and resulting into a higher level of OOPE than their counterparts [60–63].

There were noticeable differences in healthcare spending on the basis of economic status (richest /poorest) and social group affiliation (others /STs) among *Category A* states. In *Category B* states, inequalities are moderate among the above mentioned socio-economic groups. All these states (Karnataka, Andhra Pradesh, Tamil Nadu, Andaman and Nicobar, Lakshadweep, J&K, Delhi, Uttarakhand, Haryana, Himachal Pradesh, Rajasthan, Uttar Pradesh, Madhya Pradesh, Gujarat, Tripura and West Bengal) are middle-income states [11, 63, 64].

Economic status and social group affiliation were important determinants of healthcare spending among C*ategory A and B* states [35, 55, 64]. Among *Category C* states (Dadra Nagar Haveli, Daman & Diu, Sikkim, Nagaland, Assam, Manipur, Meghalaya, Mizoram, Arunachal Pradesh, Bihar, Jharkhand, Chhattisgarh and Orissa) the gap between the rich and the poor, and the disadvantaged social groups and others, is comparatively lower than it was in other categories of states [25]. In the Indian context, income, class, caste and wealth quintile are considered the most powerful stratification variables in assessing socio-economic inequalities [65].

Multivariate GLRM analysis shows that in *Category A* and *B* states, the role of religious affiliation and caste system is comparatively less important, while economic status is considered an important determinant of OOPE. Opposite trends have been observed in *Category* C states where the role of caste system continues to be a predominating factor and significantly influences the spending pattern on health. This study brings into focus that even in the 21^st^ century, with all medical advancements and institutional reforms, social institutions still have a significant influence on the healthcare seeking behaviour and spending patterns of households [66, 67]. In the Indian context, religion and caste significantly influence spending pattern among households. Results indicate significant variations in household expenditure on the basis of caste, especially among the STs [33, 34].

Similar observations have been recorded for C*ategory B* states where again economic status and social group affiliation are very important contributing factors of OOPE. Here also class concept (Economic status) is more dominant than caste but place of residence does not play an important role [27, 68]. In *Category C* states, religious affiliation and economic status both are considered equally important determinants of spending on health. Also, social group and place of residence are important determinants of OOPE among *Category C* states.

In line with the Anderson model, our study also highlights the importance of predisposing, enabling and need factors. As per the categorization of states, the role of these factors in determining the level of OOPE varies significantly in the Indian context. Among *Category A* states, enabling factors play a more dominant role than predisposing and need variables. A similar pattern was also observed among C*ategory B* states. In *Category C* states, *predisposition* to use the services such as, caste and residence, still play a major role in the determination of the level of OOPE on healthcare than other factors such as, enabling and need factors [69].

There is also evidence of the geographical concentration of the states on the basis of socio-economic inequalities and OOPE. Especially, C*ategory C* states are geographically concentrated more either on the north-eastern or southern eastern side of the country. All these states, which fall in *Category* C on the basis of OOPE, are backward states in terms of per capita income as well [70–72]. While examining healthcare spending pattern and level of OOPE as per geographic concentration, it is not easy to state whether these variations are due to geographical factors or not. These variations may be due to differences in health-seeking behaviour among the population, and to some extent, the accessibility and availability of services. Instead, socioeconomic factors affect the need for health care and are more accountable for the variations in the level of OOPE [73].

## Conclusion

In this study, we have assessed the level of OOPE, and the socio-economic and regional variations, among all Indian states and UTs. In a developing country like India, where majority of the population spends on healthcare services from their own pockets, higher government spending on health is essential. This study brings into focus healthcare inequalities in India that are based on caste and social groups. The pattern of healthcare expenditure shows large variations in access to quality healthcare by the diverse socio-religious groups in the country. Special focus must be given to financing the health care needs of the disadvantaged sections of the population, as health expenses can push these households into greater risk of poverty through mobilizing funds to cater their healthcare needs. However, designing a financial protection mechanism requires a deeper understanding of both the absolute and relative amounts of the financial burden of OOPE on the households.

There is evidence of regional disparities in terms of the level of OOPE among Indian states. Developed states are spending more on healthcare and backward states are spending less. There is a need to look into why these backward states lag behind their counterparts. Our study brings into focus the issue of inter-state differentials in OOPE causing geographic variation and concentration in healthcare spending. It has been observed that the spending on healthcare was comparatively lower among all the backward or isolated states. Overall social security measures should be enhanced, but at the same time, more priority should be assigned to these disadvantaged states to reduce the burden of OOPE, looking at the regional differences. However, geographical differences cannot explain the OOPE differentials properly and more research is needed to understand why such variation occurs and what efforts are required to address these issues.

Any resulting policy changes should reflect the needs of the backward states and local communities. It was observed that there was an association between the prevalence of socio-economic inequalities and average monthly OOPE. Policy interventions are required from both centre and the states to increase budget allocation for health spending and for reducing the level of OOPE. We hope the findings of our study will be useful for policy makers, researchers and other stakeholders to formulate appropriate strategies for removing regional imbalances in terms of health spending among Indian states.

## Limitations of the study

This study tries to analyse only the absolute burden of healthcare spending by taking into consideration various socio-economic covariates. A limited number of indicators have been taken into consideration due to data limitation, as the source data does not provide information on other important covariates. If other variables are also taken into consideration then results may vary.

## Appendix

**Table 4:** Average monthly per capita OOPE (In Rs.), average OOPE Share (%) to total and non-food expenditure by rural and urban areas of major Indian states, CES 2009–10

States	Rural	Urban	All India
	Average per capita OOPE (In Rs.)	Average per capita OOPE (In Rs.)	Average per capita OOPE (In Rs.)
J & K	66	50	62
Himachal Pradesh	89	115	92
Punjab	143	123	136
Chandigarh	25	129	113
Uttaranchal	68	98	76
Haryana	83	79	82
Delhi	18	55	53
Rajasthan	48	73	54
Uttar Pradesh	63	74	66
Bihar	26	53	29
Sikkim	11	17	13
Arunachala Pradesh	41	66	47
Nagaland	13	14	14
Manipur	18	25	21
Mizoram	18	33	25
Tripura	56	121	67
Meghalaya	19	34	22
Assam	18	37	20
West Bengal	47	136	69
Jharkhand	27	42	31
Orissa	34	69	39
Chhattisgarh	31	62	36
Madhya Pradesh	44	84	54
Gujarat	48	120	75
D & D	15	28	21
Dadra & Nagra Haveli	2	11	4
Maharashtra	78	136	102
Andhra Pradesh	63	99	73
Karnataka	38	78	52
Goa	109	114	110
Lakshadweep	73	89	82
Kerala	179	198	184
Tamil Nadu	71	105	86
Pondicherry	96	126	116
Andaman and Nicobar	34	109	63
India	57	99	68

Source: compiled from NSS CES Report (2009–10); computed by author using the unit level data records of CES 2009–10

**Table 5:** Average monthly per capita medical institutional (In Rs.), non-institutional and total health expenditure by rural and urban areas of major Indian states, CES 2009-10

States	Rural			Urban			All India		
	Per capita institutional expenditure	Per capita non-institutional expenditure	Per capita total health expenditure	Per capita institutional expenditure	Per capita non-institutional expenditure	Per capita total health expenditure	Per capita institutional expenditure	Per capita non-institutional expenditure	Per capita total health expenditure
J & K	29	37	66	9	41	50	24	38	62
Himachal Pradesh	32	58	90	41	74	115	33	59	92
Punjab	59	85	144	35	87	123	51	86	136
Chandigarh	0	26	26	50	79	129	43	71	113
Uttaranchal	19	49	68	37	61	98	24	52	76
Haryana	33	50	83	27	52	79	31	50	82
Delhi	0	19	19	25	30	55	23	29	53
Rajasthan	15	33	48	20	53	73	16	38	54
Uttar Pradesh	18	46	64	20	55	74	18	48	66
Bihar	3	23	26	9	44	53	4	25	29
Sikkim	2	10	12	1	16	17	1	11	13
Arunachala Pradesh	16	26	42	27	38	66	18	28	47
Nagaland	2	11	13	2	12	14	2	11	14
Manipur	4	15	19	7	18	25	5	15	21
Mizoram	2	16	18	6	28	33	4	21	25
Tripura	14	42	57	24	97	121	16	51	67
Meghalaya	5	15	20	7	27	34	5	17	22
Assam	4	15	19	8	29	37	4	16	20
West Bengal	12	36	48	45	90	136	20	49	69
Jharkhand	7	21	28	8	35	42	7	24	31
Orissa	6	28	34	22	47	69	8	31	39
Chhattisgarh	5	25	31	25	37	62	9	27	36
Madhya Pradesh	9	35	44	22	61	84	12	41	54
Gujarat	28	20	48	65	55	120	42	33	75
D & D	5	10	15	14	14	28	9	12	21
Dadra and Nagra Haveli	0	2	2	7	4	11	2	2	4
Maharashtra	32	46	78	55	81	136	42	60	102
Andhra Pradesh	18	45	63	28	71	99	21	52	73
Karnataka	10	28	38	28	50	78	16	36	52
Goa	23	86	109	39	75	114	27	83	110
Lakshadweep	57	17	73	64	25	89	60	21	82
Kerala	70	109	179	78	120	198	72	112	184
Tamil Nadu	20	50	71	28	76	105	24	62	86
Pondicherry	19	77	96	23	103	126	22	94	116
Andaman and Nicobar	25	9	34	81	28	109	46	16	63
India	18	39	57	34	65	99	22	46	68

Source: compiled from NSS CES Report (2009–10); computed by author using the unit level data records of CES 2009–10

## Abbreviations

CES: Consumer Expenditure Survey
GLRM: Generalized Linear Regression Model
GoI: Government of India
ILO: International Labour Organization
J&K: Jammu and Kashmir
LMICs: Low- and Middle-Income Countries
MoSPI: Ministry of Statistics and Programme Implementation
NSSO: National Sample Survey Organization
NCDs: Non-communicable diseases
OLS: Ordinary Least Square
OBC: Other Backward Caste
OOPE: Out of Pocket Expenditure
SC: Scheduled Caste
ST: Scheduled Tribe
UTs: Union Territories
UHC: Universal Health Coverage
WHO: World Health Organization

## Footnotes

1 OOPE are non-reimbursable fees which a patient or family is responsible for paying directly to health practitioners or suppliers, without intervention of a third party.

2 Informal sector economic activities include all forms of non-standard atypical, alternative, irregular and precarious forms of work, and includes all persons in employment who, by law or in practice, are not subject to national labour legislation, income taxation, social protection or entitlement to employment benefits (International Labour Organization (ILO), 2012).

3 Inpatient expenditure comprises of health care services delivered to patients who are formally admitted to hospitals, ambulatory premises or self-standing centers.

4 Outpatient expenditure includes expenses on physician’s private office, hospital outpatient center or ambulatory-care center. Patient does not stay overnight.

5 The Scheduled Castes (SCs), Scheduled Tribes (STs) and Other Backward Class (OBC) indicate constitutional classification of various groups of historically disadvantaged indigenous people in India. Various provisions are made by the Constitution of India, to provide reservation, protection and safeguards, in public employment in respect of the persons belonging to the SCs/STs and OBCs.

6 The *Scheduled Castes* are sometimes referred to as lower caste or Dalits, and are officially regarded as the socially disadvantaged group of population.

7
*Scheduled Tribes* is used as an official term for Adivasi or indigenous people, mainly residing in the hilly, forest or other inaccessible areas. In the Indian context, these people are mainly the indigenous people who are officially regarded as socially disadvantaged.

8 Other Backward Class (OBC) is a collective term used by the Constitution of India to categorize castes which are socially and educationally disadvantaged.
